# Implicit Learning of Arithmetic Regularities Is Facilitated by Proximal Contrast

**DOI:** 10.1371/journal.pone.0048868

**Published:** 2012-10-31

**Authors:** Richard W. Prather

**Affiliations:** Department of Psychological and Brain Sciences, Indiana University, Bloomington, Indiana, United States of America; Utrecht University, The Netherlands

## Abstract

Natural number arithmetic is a simple, powerful and important symbolic system. Despite intense focus on learning in cognitive development and educational research many adults have weak knowledge of the system. In current study participants learn arithmetic principles via an implicit learning paradigm. Participants learn not by solving arithmetic equations, but through viewing and evaluating example equations, similar to the implicit learning of artificial grammars. We expand this to the symbolic arithmetic system. Specifically we find that exposure to principle-inconsistent examples facilitates the acquisition of arithmetic principle knowledge if the equations are presented to the learning in a temporally proximate fashion. The results expand on research of the implicit learning of regularities and suggest that contrasting cases, show to facilitate explicit arithmetic learning, is also relevant to implicit learning of arithmetic.

## Introduction

In many learning domains behavioral researchers have sought to characterize the knowledge that separates the novices from the experts, and to describe how that knowledge is acquired. Learners have been shown to use principles in a variety of domains. Principles are defined as regularities or general rules within a particular learning domain. Research of learners principle knowledge includes areas such as counting [1], proportional reasoning [2], artificial grammar learning [3], [4], physics [5] and language acquisition [6]. In arithmetic the relation between operands in simple equations is such a principle. For addition equations with natural numbers (A+B = C) the sum (C) must be greater that either operands, A and B. In the domain of arithmetic the majority of research concentrates on determining which principles learners know at particular points in development [7]–[14]. This type knowledge inventory is both useful and necessary. However, it leaves a crucial questions unanswered: what types of experiences facilitate learning? Only recently have studies begun to address the learning of arithmetic principles [15]–[20]. Prior research [18] has suggested that children can learn arithmetic principle knowledge through exposure to a mix of principle-consistent and principle-inconsistent equations. The current study expands on these initial investigations of arithmetic principle acquisition, drawing on what is known from psychological research about the implicit learning of regularities. Specifically, we evaluate the effect of temporal proximity in contrasting equation examples of different types. Though prior work focuses on child participants in a classroom setting, in the current study we evaluate adult participants in a lab setting. This enables us to evaluate how similarly arithmetic principles may be implicitly learned in comparison to the expansive literature on implicit learning of artificial grammars.

### Implicit learning of regularities

Though the current research focuses on arithmetic principles, findings regarding principle learning in other domains (e.g., language acquisition, counting) are informative. Research on implicit learning of artificial grammars is particularly relevant as researchers often examine participants' learning of regularities [21]. In a typical artificial grammar learning study, participants are given a large amount of exposure to examples that correspond to a particular grammar [3], [4], [22]–[24]. These grammars are generally strings of letters of varying length, such as ABFE or ABBQW, that correspond to a predetermined finite state grammar. The participant may be instructed to memorize the examples or otherwise pay attention to them, and is not told that there are any regularities in the stimuli to learn. After the initial exposure, participants are told that regularity was present, and their knowledge of the regularity is assessed via their evaluation of novel examples that either violate or are consistent with the regularity. The general consensus of this line of research is that participants are often able to learn complex regularities. *Thus, participants may be able to learn regularities in a domain through exposure to examples*.

These findings suggest that it may be effective to investigate the learning of arithmetic principles through the manipulation of the types of example arithmetic equations to which learners are exposed. It is certainly the case that arithmetic learners see many examples of arithmetic equations inside and outside of formal education settings. Thus determining whether arithmetic principles may be learned in this way would be particularly useful. The current study addresses two factors, exposure to principle-inconsistent examples and the temporal proximity of said examples.

#### Experience with principle-inconsistent examples

Learning of principles in artificial grammar and language studies generally involves learning by positive examples only [3], [25]. Participants in these studies generally only view examples that are consistent with the principle during training. The current study considers a mix of principle-consistent and principle-inconsistent examples. The idea is that a mix of principle-consistent and principle-inconsistent equations will highlight the relevant regularities for the learner. Prior research of arithmetic knowledge suggests that *contrast* between principle-consistent and principle-inconsistent examples may highlight regularities across examples within a domain [26], [27]. These results suggest that a training task that includes both principle-consistent and principle-inconsistent examples may facilitate acquisition of arithmetic principles.

#### Temporal proximity of principle-inconsistent examples

Principle learning may be facilitated by input that highlights the regularity repeatedly within a small amount of time. Prior work suggests that in order to learn arithmetic principles, the learner needs to have the relevant regularity highlighted repeatedly within a short time span [14], [16], [20]. Thus examples presented to participants are most effective when blocked together. Once a particular regularity is highlighted sufficiently, the learner integrates that regularity into their representation of the domain. *Repeated temporally proximal examples that highlight a specific arithmetic principle should lead to the acquisition of that principle by the learner*. These findings suggest that input that highlights a particular regularity must be presented to the learner in a particular sequence in order to be effective. Prior work of arithmetic principle learning that suggests temporal proximity is effective unfortunately does not control for the number of stimuli presented. Thus it is impossible to determine to what degree learning is due to the blocked nature of the stimulus presentation as opposed to the amount of stimuli presented to the learner. The current study will disentangle these factors and determine the degree to which the temporal proximity of example presentation affects learning.

### What are the correlates of principle knowledge?

A secondary goal this study is to characterize the correlates of arithmetic principle knowledge acquisition. Knowledge of arithmetic principles may correlate with learning processing of arithmetic equations and their performance in arithmetic.

#### Associations between principle knowledge and equation encoding

Prior research has attributed changes in learners' knowledge to changes in their *encoding* (or *representation*) of the problem domain [16], [18], [28]–[31]. Encoding refers to the fact that for a given stimulus, whether it is a simple arithmetic equation, a chessboard scene or a physics problem, there are certain characteristics that can be attended to. Consider the example of a simple equation, *8+3 = 11*. There are many characteristics that could be noted: the color of the numerals, the order of the numbers, the type of operation, the value of the operands, relative spacing of the digits, the relative magnitude of the operands. This study will address the hypothesis that increased principle knowledge will be associated with change in equation encoding.

Consider learners' encoding of simple arithmetic equations. The learner may note characteristics of the equation such as the values of the operands, the operation, the order of the operands, specific characteristics of the operands such as evenness or oddness, and so forth. The learner's encoding of the equation involves noting and prioritizing a set of characteristics. Some characteristics may be deemed very important while others are virtually ignored.

#### Association between principle knowledge and arithmetic skill

What the learner attends to can have consequences for performance, for example the relative spacing of digits can influence performance on arithmetic tasks [32]. For example, children have been shown to have difficulty solving equations in the form *A+B+C = A+__*. Learners who had difficulty solving these types of equations also showed poor encoding of the equations [30]. A learner's representation of a particular problem may draw, not only on information encoded from the given problem, but also on other sources of knowledge from long-term memory, such as prior knowledge of common problem schemas [33].

Past research suggests that principle knowledge contributes to expertise in a domain [5], [34]. Previous work [19] has shown connections between learners' problem representation and their principle knowledge. These types of connections have been explored on occasion in the literature, largely using correlational designs [10], [13], [35], [36]. This study will investigate connections between principle knowledge and arithmetic problem solving. A key difference between this research and prior work is that we will test a more causal connection between principle knowledge and problem solving by manipulating principle knowledge and examining effects on problem solving. Thus, this research will seek to show that acquiring arithmetic principles leads to improved problem solving, supporting the oft-held claim that principle knowledge is a crucial aspect of arithmetic knowledge.

## Methods

The current study focuses on the *Relationship to Operands* principle (Dixon et al., 2001). Generally stated, this principle describes the relationships between the operands and the result in a given arithmetic equation. The exact relationship varies depending on the operation. For natural numbers in simple addition equations (A+B = C), the sum (C) must be greater than the two addends (A and B). In simple subtraction equations (A–B = C), the difference (C) must be less than the subtrahend (A), however it may have any relationship with the minuend (B). In simple multiplication equations (A×B = C), the product (C) must be greater than both operands (A and B). In simple division equations (A÷B = C), the quotient (C) must be smaller than the dividend (A), however it may have any relationship with the divisor (B). These are all considered examples of the *Relationship to Operands* principle, though the details for each operation differ slightly.

In the case of the relationship to operands principle, the characteristic of the arithmetic equation that may be crucial to encode is the relative magnitude of the operands and the result. Learners whose encoding prioritizes the relative magnitude of the operands and result will demonstrate principle knowledge. A learner who does not prioritize the relative magnitudes of the operands and result is unlikely to show knowledge of relationship to operands.

### Ethics Statement

The research procedures described below were completed in accordance with approval from the Institutional Review Board at the University of Wisconsin – Madison. Written consent was obtained from all participants prior to testing.

### Participants

Adult participants (*n* = 119) were recruited through the University of Wisconsin-Madison introductory psychology participant pool. Participants received extra credit for their participation. Participants were randomly assigned to the experimental conditions.

### Apparatus

Experimental stimuli were presented either on paper or via computer, depending on the task. Computer presentations used the PsyScope 1.2.5 interactive graphic system for experimental design and control [37].

### Procedure

#### Assessments of principle knowledge

The experiment used a multifaceted approach to characterizing principle knowledge [38], [39]. The tasks used for knowledge assessment give learners several ways to display principle knowledge. The overall procedure included 1) initial completion of two principle knowledge assessments, the Equation Evaluation task and the Equation Verification task, 2) a training task, 3) a second completion of the Equation Evaluation and Equation Verification tasks, 4) assessments of equation encoding and 5) arithmetic problem solving. Both the Equation Evaluation and Equation Verification tasks are completed twice by each participant, using different stimulus sets.

##### Equation Evaluation task

In this type of assessment the learner is given the opportunity to differentiate between examples that violate a principle and those that do not. Participants may indicate that equations that violate a principle are “worse” or “more wrong” than equations that do not. The evaluation task was based on a task used in prior research [12] to assess knowledge of principles. Participants viewed sets of solved division equations, presented one at a time on sheets of paper. Each set consisted of eight equations presented in a matrix. Participants were told that each set had been solved by “a hypothetical student who is learning arithmetic” (see Appendix). Participants were also told that though all of the equations were incorrect, they might believe that some students made better attempts at arithmetic than others. Participants were asked to rate each attempt on a scale from 1 to 7, with 1 indicating very bad and 7 indicating pretty good. This task was not timed.

Sets of equations were constructed in pairs. Each pair contained the same operands for each of the eight equations but varied in the answers. Answers were controlled for so that the average deviation from the correct answer was the same across both sets, and so that one set contained one principle principle-inconsistent, while the other contained no principle-inconsistent equations. Since the average deviation from the correct answer was controlled for participants could not differentiate equation sets based solely on answer plausibility. Participants were inferred to have knowledge of the principle if they rated principle-inconsistent sets lower than principle-consistent sets.

##### Equation Verification task

Participants viewed solved arithmetic equations (80 total) presented serially on a computer monitor, and were asked to judge whether each equation was correct (e.g., “54÷6 = 9”) or incorrect (e.g., “15÷5 = 2”). Participants were asked to respond as quickly and accurately as they could. Both speed and accuracy were recorded. The task can be performed by calculating the answer to each equation and comparing it to the presented answer. Participants likely used this strategy on many trials. However, a subset of trials can be solved via a “short cut”. Any equation that violates a principle can be verified as incorrect without calculation. For example, 225÷15 = 345 can be quickly dismissed by noting that 345>225 (in contrast, try 225÷15 = 13). Equations were selected to control for deviation from the correct answer. We took as an indication of participants' principle knowledge if they used significantly less time to reject violation equations as compared to principle-inconsistent equations.

Though both the equation evaluation task and verification task were designed to assess principle knowledge they use different types of evidence. The verification task is based on the application of procedures while the evaluation task is based on evaluation of examples. These two types of knowledge assessments do not necessarily yield similar results [18], [38].

##### Training task

Participants' experience was manipulated using a training task similar to the methods in typical artificial grammar learning experiments [21]. Training conditions differed based on the proportion of principle principle-inconsistent equations used and in the temporal proximity of their presentation. Comparisons between the pre-to-post gain scores of participants across conditions indicate whether experience with principle consistent and principle-inconsistent equations affects learning.

The training task involved serial presentation of arithmetic equations on a computer screen. Participants were instructed that they were to view a display of equations solved by two students and to decide which student understood arithmetic better. Each equation was presented for 2000 ms. Equations' solutions were marked by color as correct (green) or incorrect (red). There were a total of 120 equations split between the two students. Equations were presented in blocks of 30 alternating by student.

Equation sets included a mix of correct, incorrect principle-consistent and incorrect principle-inconsistent equations. The proportion and placement of the principle-inconsistent equations depended on the condition to which the participant was assigned. The exact set of equations viewed by participants varied by condition. There were two factors manipulated in a 2×2 factorial design: number of principle-inconsistent equations (high, low) and temporal proximity of principle-inconsistent equations (blocked, interleaved). Thus the four conditions were as follows: high-blocked( n = 22), high-interleaved (n = 23), low-blocked (n = 21), and low-interleaved (n = 21).

In the high principle-inconsistent conditions there were 12 principle-inconsistent, 10 correct and 8 principle-consistent equations (40% principle-inconsistent) for each block. In the low there were 6 principle-inconsistent, 10 correct and 14 principle-consistent equations (20% principle-inconsistent) for each block. In both conditions violation equations are blocked such that they all appear in succession in the middle of the presentation. For high and low interleaved conditions the proportion of equations types is the same as previously stated. However, for interleaved conditions violation equations never appeared in succession, instead a principle-inconsistent or correct equation always followed.

In addition to these conditions, there was also group of participants (n = 32) who viewed no principle-inconsistent equations at all. In this condition, all of the equations in the training stimuli were consistent with the arithmetic principle; this included 10 correct and 20 incorrect principle-consistent equations. Thus, the full design was a 2×2 factorial design, with one additional control condition.

##### Equation encoding task

To address the possibility that improvements in equation encoding lead to principle knowledge, a task was devised to characterize learners' equation encoding. Assessments of equation encoding have been used in other research that investigates arithmetic knowledge [30]. This includes a reconstruction task in which learners needed to recreate equations from memory. Learners who fail to encode a particular aspect of an equation, or who encode it poorly, will tend to reproduce that aspect incorrectly. This study employs a similar logic in assessing encoding.

Participants viewed equations presented briefly (990 ms) on a computer screen. After each equation the participant saw a series of four letters. The participant was then asked to answer a question about the letters (e.g., *Was the first letter a z?*) and then to answer a question about the equation. Questions about the equation were of four types: (a) identity (e.g., *Was the first number 27?*), (b) relationship (e.g., *Was the first number bigger than the third?*), (c) operation (e.g., *Was the operation division?*) or (d) parity (e.g., *Was the third number odd?*). The question types were mixed throughout the trials; thus, for any given trial the participant did not know which type of question would be presented.

##### Word problem task

Prior research suggests that arithmetic principle knowledge may correlate with word problem skills [19]. In order to investigate the possible consequences of arithmetic principle knowledge we used a word problem task. Participants viewed story scenario problems such as “*Alayna is collecting CDs. She picked out 5 CDs of the same price. All the CDs were on sale for 7 dollars off. Alayna paid 14 dollars for each CD. How much would she have spent altogether, if the CDs had not been on sale*?” In each case participants selected from several options an equation that represented the given scenario, and that could be solved for the unknown (see Appendix). This task included 5 problems that involved division and 5 that involved multiplication.

## Results

### Did participants' principle knowledge change with training? If so, what factors affect learning?

To compare principle knowledge before and after the training task, a “learning” score was calculated for each participant. This was done by comparing the difference in ratings of principle-consistent and principle-inconsistent sets in the evaluation task pre and post training. For example, if a participant rated principle-consistent equations higher than principle-inconsistent equations by 0.05 on the pre-training evaluation task and by 0.70 on the post-training evaluation task, that participant's learning score would be 0.65. The data analysis compares learning scores across conditions.

**Figure 1:**
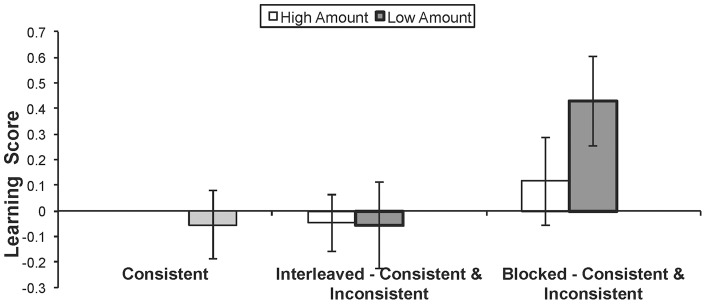
Learning scores for participants in each of the training conditions.

Recall that participants viewed input that varied along two factors, number of principle-inconsistent equations (high vs. low), and proximity (blocked, interleaved). we conducted a 2 (number of principle-inconsistent equations: high or low) x 2 (proximity: blocked or interleaved) ANOVA with learning scores as the dependent measure. The data are presented in Figure 1. A significant effect of proximity was found, F(1, 83)  = 4.21, p = .04, η^2^ = 0.046. Participants in the blocked conditions had higher learning scores (M = .269) than participants in the interleaved conditions (M = −0.052). Number of principle-inconsistent equations did not significantly affect improvement scores, F(1, 83)  = .919, p = 0.34. Participants in the high-number conditions did not have significantly different learning scores from participants in the low-number conditions (Ms  = 0.032 and 0.186, respectively). The interaction of the two factors was also not significant, F(1, 83)  = 1.04, p = 0.31. *In sum, improvement on the evaluation task was dependent on the temporal proximity of the principle-inconsistent equation, not the number of principle-inconsistent equations.*

To examine whether principle-inconsistent equations facilitated learning more than principle-consistent examples, we performed a planned comparison between the blocked principle-inconsistent group and the bocked principle-consistent group. This comparison was marginally significant, t(73)  = 1.89, p = .061, *d* = 0.39.

We also examined whether number of principle-inconsistent equations affected learning within the blocked conditions. The difference in learning scores between the high-blocked and low-blocked conditions was not significant, t(41)  = 1.26, p = 0.21.

The verification task was also used as a measure of principle knowledge. For this task there were two analyses done, reaction time and accuracy. For each dependent measure, reaction time and accuracy, we conducted a 2 (number of principle-inconsistent equations: high or low) x 2 (proximity: blocked or interleaved) ANOVA. For both analyses there were no significant effects of amount, proximity or their interaction; reaction times, number F (1, 83)  = 1.35, p = .24, proximity F (1, 83)  = 0.36, p = 0.54, number by proximity interaction F (1, 83)  = 1.98, p = 0.16; accuracy, number F (1, 83)  = 2.38, p = .12, proximity F (1, 83)  = 0.20, p = 0.65, number by proximity interaction F ( 1, 83)  = 1.72, p = 0.19.

### Were there correlates to having principle knowledge?

#### Equation Encoding task

The most relevant comparison is between learners' post-training principle knowledge and their encoding scores. Encoding scores were calculated for each of the four types of questions in the encoding task: relationship, identity, parity and operation. Scores were based on the number of trials answered correctly.

Based on participants' post-training principle knowledge scores, participants were divided into three equal size groups, with high, medium and low principle knowledge. For these groups, mean scores on the post-training evaluation task were 1.14, 0.32, and −0.29, respectively. For each of the four encoding question types, we ran a one-way ANOVA and conducted post hoc comparisons.

For both relationship and identity encoding scores, the overall ANOVA was significant, F(1, 116)  = 3.66, p = .03, η^2^ = 0.059 and F(1, 116)  = 5.94, p = .003, η^2^ = 0.093, respectively. For both relationship and identity encoding scores, participants in the low principle knowledge group performed significantly more poorly than participants in the medium or high knowledge groups. For relationship encoding scores, the low knowledge group (M = 0.71) scored significantly lower than the medium group (M = 0.81, p = .023, *d* = 0.586), though not significantly lower than the high group (M = 0.78, p = .21). For identity encoding scores, the low knowledge group (M = 0.63) scored significantly lower than both the medium group (M = 0.73 p = .05, *d* = 0.472) and the high group (M = 0.78, p = .003, *d* = 0.842). It is worth noting that if the identity of the numbers is accurately encoded, the relative magnitude of the operands and the result can be inferred. Thus, it is not surprising that the question types yielded similar results.

Neither parity nor operation encoding scores yielded significant results, F(1, 116)  = 0.384, p = 0.68 and F(1, 116)  = 0.087, p = 0.91. Thus, there were no significant differences between the principle knowledge groups in encoding the parity of the numbers or the operations used in the equations. Note that the parity of the numbers is not relevant to the relationship to operands principle. Further, regardless of which operation is involved, the relative magnitudes of the operands and result are crucial to encode; thus, variations in encoding operation as a function of principle knowledge were also not expected.

These results of this task suggest that learners with greater arithmetic principle knowledge encode specifically the relative magnitudes and the identities of the numbers in arithmetic equations better than learners with less principle knowledge.

#### Word problem task

Because the training was in division only, we examined participants' performance on the division items on the word problem task. We conducted a 2 (number of principle-inconsistent) x 2 (proximity) ANOVA with division sub-scores as the dependent measure. Neither factor was significant, nor was the interaction, proximity F(1, 83)  = 2.39, p = 0.12, number of principle-inconsistent equations F(1,83)  = 0.37, p = 0.54, number by proximity interaction F (1, 83)  = .34, p = .55. Participants in the blocked conditions were correct on 78% of the division items while participants in the interleaved condition were correct on 71% of the items.

## Discussion

The current results demonstrate that increases in arithmetic principle knowledge are related to exposure to temporally proximal principle-inconsistent equations in addition to principle consistent equations. This result is unexpected based on much of the implicit learning literature, which generally focuses on learning via principle-consistent examples only. This suggests that the learning of arithmetic principles may occur via a different mechanism. Since exposure that includes multiple types of examples seems to work best, it is possible that it is the action of contrasting between the types that facilitate learning of principles. Previous work in arithmetic learning suggest that contrasting cases is beneficial to mathematical conceptual and procedural knowledge [26], [40].

The results of this study also suggest that learners with relatively low principle knowledge were also poor at encoding the relative magnitudes of the numbers in the equations and the identity of the numbers in the equations. Although accurate equation encoding may be required for knowledge of the principle, it does not seem to be the case that accurate encoding automatically leads to principle knowledge. This is suggested by the fact that high principle knowledge participants did not encode the problems significantly more accurately than the medium principle knowledge group. Consistent with prior [18] work results suggest that accurate encoding is necessary but not sufficient, for principle knowledge.

### Conclusions

This research investigated how learners acquire knowledge of arithmetic principles though structured input. We addressed both the possible mechanism of change in encoding and the types of experience that may facilitate that change. The results of this study suggest that equation encoding is indeed related to arithmetic principle knowledge. We initially hypothesized a direct relationship between encoding of relative magnitudes and knowledge of relationship to operands. The results suggest that this relationship may not be as straightforward as originally hypothesized; there may be other mediating factors.

Finally, the results suggest that structuring the types of examples so as to give the learner an opportunity for contrast may facilitate learning of arithmetic principles; specifically, a mix of correct, principle-consistent and principle-inconsistent equations seems beneficial. This result may have some application to formal educational settings. While it is certainly the case than explicit instruction can play a role in formal education, learners have much more experience and opportunity for implicit leaning than explicit formal instruction. Prior research in contrasting cases [26], [40] suggests that this strategy may be useful in increasing children's knowledge of mathematics.
